# dbOGAP - An Integrated Bioinformatics Resource for Protein O-GlcNAcylation

**DOI:** 10.1186/1471-2105-12-91

**Published:** 2011-04-06

**Authors:** Jinlian Wang, Manabu Torii, Hongfang Liu, Gerald W Hart, Zhang-Zhi Hu

**Affiliations:** 1 Department of Oncology, Georgetown University Medical Center, 3300 Whitehaven Street, Suite 1200, NW, Washington, DC 20007, USA; 2 ISIS Center, Georgetown University Medical Center, 2115 Wisconsin Avenue, Suite 603, Washington, DC 20007, USA; 3 Department of Biostatistics, Bioinformatics and Biomathematics, Georgetown University Medical Center, 4000 Reservoir Road, Building D, Suite 180, NW, Washington, DC 20057, USA; 4 Biological Chemistry, School of Medicine, Johns Hopkins University, 725 N. Wolfe Street, 515 WBSB, Baltimore, MD 21205, USA

## Abstract

**Background:**

Protein O-GlcNAcylation (or O-GlcNAc-ylation) is an O-linked glycosylation involving the transfer of β-*N*-acetylglucosamine to the hydroxyl group of serine or threonine residues of proteins. Growing evidences suggest that protein O-GlcNAcylation is common and is analogous to phosphorylation in modulating broad ranges of biological processes. However, compared to phosphorylation, the amount of protein O-GlcNAcylation data is relatively limited and its annotation in databases is scarce. Furthermore, a bioinformatics resource for O-GlcNAcylation is lacking, and an O-GlcNAcylation site prediction tool is much needed.

**Description:**

We developed a database of O-GlcNAcylated proteins and sites, dbOGAP, primarily based on literature published since O-GlcNAcylation was first described in 1984. The database currently contains ~800 proteins with experimental O-GlcNAcylation information, of which ~61% are of humans, and 172 proteins have a total of ~400 O-GlcNAcylation sites identified. The O-GlcNAcylated proteins are primarily nucleocytoplasmic, including membrane- and non-membrane bounded organelle-associated proteins. The known O-GlcNAcylated proteins exert a broad range of functions including transcriptional regulation, macromolecular complex assembly, intracellular transport, translation, and regulation of cell growth or death. The database also contains ~365 potential O-GlcNAcylated proteins inferred from known O-GlcNAcylated orthologs. Additional annotations, including other protein posttranslational modifications, biological pathways and disease information are integrated into the database. We developed an O-GlcNAcylation site prediction system, OGlcNAcScan, based on Support Vector Machine and trained using protein sequences with known O-GlcNAcylation sites from dbOGAP. The site prediction system achieved an area under ROC curve of 74.3% in five-fold cross-validation. The dbOGAP website was developed to allow for performing search and query on O-GlcNAcylated proteins and associated literature, as well as for browsing by gene names, organisms or pathways, and downloading of the database. Also available from the website, the OGlcNAcScan tool presents a list of predicted O-GlcNAcylation sites for given protein sequences.

**Conclusions:**

dbOGAP is the first public bioinformatics resource to allow systematic access to the O-GlcNAcylated proteins, and related functional information and bibliography, as well as to an O-GlcNAcylation site prediction tool. The resource will facilitate research on O-GlcNAcylation and its proteomic identification.

## Background

O-GlcNAcylation, or O-GlcNAc-ylation to distinguish it from acylation, is an O-linked glycosylation involving the β-attachment of a single N-acetylglucosamine (GlcNAc) to the serine (Ser)/threonine (Thr) residues catalyzed by O-GlcNAc transferase (OGT), whose removal is catalyzed by O-GlcNAcase (OGA) [1]. The two O-GlcNAc cycling enzymes OGT and OGA are each encoded by a single gene in mammalian species. Unlike N-linked or mucin-type O-linked glycosylation, O-GlcNAcylation occurs primarily in nucleocytoplasmic proteins [1]. Analogous to phosphorylation, the modification is dynamic and the O-GlcNAc moiety is not further extended [1]. O-GlcNAcylation is also often reciprocal to phosphorylation at the same or adjacent Ser/Thr residues [1-3], which led to a "Yin-Yang" hypothesis on protein functions modulated by the two post-translational modifications (PTMs) [4] through competitively blocking each other's occupancy at given sites. For example, reciprocal O-GlcNAcylation and phosphorylation at the same Ser16 of murine estrogen receptor β (ERβ modulate the degradation of ERβ by stabilizing or destabilizing the protein, respectively [5]. Similarly, O-GlcNAcylation of p53 at Ser149 is associated with decreased phosphorylation at the adjacent Thr155, resulting in decreased p53 ubiquitination and subsequent degradation, thus stabilizing p53 [6]. In contrast to the enormous body of research on phosphorylation, the amount of research on O-GlcNAcylation has been disproportionally small due to difficulties in detecting the O-GlcNAc group, partly because of its being labile, dynamic, and substoichiometric [7]. Over 600 proteins have been reported to be O-GlcNAcylated since it was first identified in 1984 [8], many of which were identified in recent years [1-3,9-11] as a result of improved mass spectrometry technologies. Growing evidences now suggest that O-GlcNAcylation is very common and has broad roles in physiology and diseases, especially through its reciprocal interplay with phosphorylation, e.g., regulation of insulin signaling, transcription, and roles in diabetes and neurodegenerative diseases [2].

A number of bioinformatics databases have been developed for protein post-translational modifications, including those of general PTMs, e.g., dbPTM [12], or specific types, e.g., databases of protein phosphorylation, e.g., PhosphoELM [13], PhosphoSite [14], and those of protein glycosylation [15], ubiquitination [16] and protease cleavage [17]. By contrast, there has been no special database dedicated to O-GlcNAcylated proteins and sites, and their annotations are also scarce in protein databases, e.g., only ~100 experimental O-GlcNAcylation sites for 35 proteins are currently annotated in UniProtKB [18]. Moreover, O-GlcNAcylation annotations have not been included in the specialized glycosylation databases (e.g., GlycoBase, the Functional Glycomics Gateway) [15,19].

Because of growing interests in studying the crucial roles of O-GlcNAcylation in cell signaling and many other cellular processes, identifying the site motifs and computationally predicting the O-GlcNAcylation sites become important bioinformatics tasks to assist those studies. Unlike N-linked glcycosylation with a consensus motif of "Asn-X-Thr/Ser", O-linked glycosylation, including mucin-type O-glycosylation and O-GlcNAc glycosylation, has not yet found well-defined sequence motifs. The past effort in developing prediction method for O-glycosylation has mostly focused on the mucin-type [20-23]. To our best knowledge there has been only one site prediction tool for O-GlcNAcylation, YinOYang, which is an artificial neural network system trained on sequence fragments of ~40 GlcNAcylation sites available at the time [24]. The motif of O-GlcNAcylation remains poorly defined, and there is a pressing need to develop an O-GlcNAcylation site prediction tool based on a much greater number of experimental O-GlcNAcylation sites available now.

Here we report the development of a database of O-GlcNAcylated proteins and sites (dbOGAP) for all currently known O-GlcNAcylated proteins reported from literature, and of an O-GlcNAcylation site prediction system (OGlcNAcScan) based on nearly 400 O-GlcNAcylation sites. Both the database and the prediction system are available through the dbOGAP web site, which serves as a public bioinformatics resource to facilitate research on O-GlcNAcylated proteins and to assist proteomic identification of O-GlcNAcylation sites.

## Construction and Content

### 1. The Database Development

**Figure 1:**
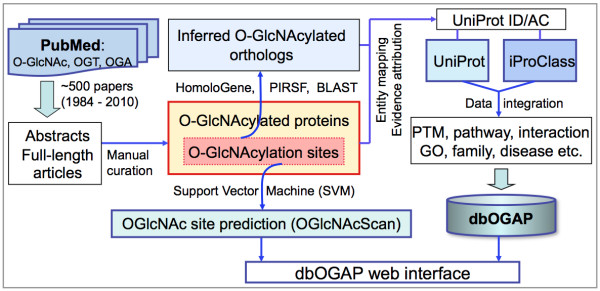
**Overall workflow of dbOGAP development**.

The primary data source used for developing the dbOGAP database is literature about O-GlcNAcylated proteins published since O-GlcNAcylation was first discovered in early 1980's [8]. Figure 1 depicts the overall workflow of the dbOGAP database and web site development. About 500 original and review articles were retrieved from PubMed (April 2010) that are related to protein O-GlcNAcylation and/or the O-GlcNAc cycling enzymes OGT and OGA. Abstracts and full-length articles were used to identify experimentally determined O-GlcNAcylated proteins and sites. The proteins were then mapped to UniProtKB entry records based on sequences and/or sequence identifiers (IDs) followed by manual verification. O-GlcNAcylated proteins and sites determined only from large-scale mass spectrometry (MS) without further validation using targeted MS and/or additional biochemical methods were annotated with evidence tags (e.g., "LS: MALDI-TOF-MS"). Orthologs of known O-GlcNAcylated proteins with identified O-GlcNAcylation sites were populated based on the HomoloGene groups [25] and/or BLAST neighbors [26], where the potential O-GlcNAcylation sites on the orthologs were inferred based on the conserved Ser/Thr residues. The experimental or inferred O-GlcNAcylation was attributed with literature (PubMed ID) or inference (from orthologs), respectively. A small number of currently annotated O-GlcNAcylated proteins in UniProtKB were also integrated into dbOGAP with the source attributed. Additional protein annotations, including other protein modifications (e.g., phosphorylation) and site features, Gene Ontology, pathways and disease information were integrated into dbOGAP from UniProtKB [18] or iProClass [27] databases.

### 2. The O-GlcNAc Site Prediction

An O-GlcNAcylation site prediction system, OGlcNAcScan, was developed based on annotated O-GlcNAcylation sites in dbOGAP using the SVM^light ^implementation of Support Vector Machine (SVM) [28]. A training data set of the prediction system consists of 373 positive instances that are experimental O-GlcNAcylation sites in 167 protein sequences from dbOGAP, and also of 29,897 negative instances that are the rest of the un-annotated Ser/Thr sites in the same protein sequences. Given a Ser/Thr site, *n *upstream and *n *downstream amino acids were regarded as its sequence context and then 2*n*+1 amino acids, including the O-GlcNAcylated Ser or Thr residue in the middle, were converted into a vector of binary values (0 or 1) using the widely-used *sparse encoding *method described, for example, in Julenius et al. 2005 [21]. Note, if the site is less than *n *amino acid away from the sequence terminals, the *end-of-sequence symbol *is padded at the terminal as many as needed to derive a fixed-length sequence fragment. In this encoding method, each amino acid type and the end-of-sequence symbol is coded with 21 binary values, e.g., 100...0 (one followed by 20 zeros) for Ala, 010...0 for Arg, ..., and 000...1 for end-of-sequence), and the resulting feature vector consists of 21 × (2*n*+1) binary values. For different values of *n*, we trained SVM classifiers with the RBF kernel. The parameters involving these classifiers, *C *and *γ*, were optimized through 5-fold *cross-validation *tests, where classifiers were trained and tested, respectively, on a four-fifths and the remaining one-fifth of the data set for five times. We explored different sequence encoding methods, such as frequencies of amino acid types [21,23] and gappy bi-grams/dimers [22], but the orthodox sparse encoding method with *n *= 5 yielded the best prediction performance.

### 3. The Database and the Web site Implementation

The dbOGAP database is implemented using the open source relational database management system, MySQL, with tables to store and manage the O-GlcNAcylation protein entries, O-GlcNAcylation sites from different sources and related literature information. The database is deployed on RedHat Enterprise Linux operating system (version 5.5). The Apache web server (version 2.2.15) (http://httpd.apache.org/) with the security enhanced module ModSecurity (version 2.5.10, http://www.modsecurity.org/), was deployed for the dbOGAP web site. All data query and retrieval from the dbOGAP web site is accomplished by scripts written in Perl, PHP and Javascript.

## Utility

### 1. The Database Contents

**Figure 2:**
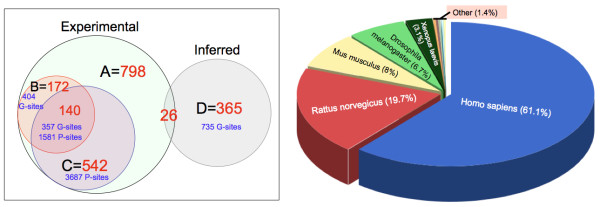
**Statistics of protein entries in dbOGAP.*** Left*, Venn diagram showing the number of O-GlcNAcylated proteins and modification sites in the dbOGAP database. There are a total of 1163 protein entries in the database. A - Experimental O-GlcNAcylated proteins; B - Proteins with identified O-GlcNAcylation sites (G-sites); C - Proteins with identified phosphorylation sites (P-sites); D - Proteins with inferred O-GlcNAcylation sites based on orthologs with known O-GlcNAcylation sites. *Right*, Taxonomic distributions of experimentally determined O-GlcNAcylated proteins in dbOGAP. Numbers shown in the pie chart are percentage of proteins in given species over the total number of proteins in the database. "Other" species include *Bos taurus *(3 protein entries), *Gallus gallus *(2), *Coturnix coturnix japonica *(1), *Rhea americana *(1), *Macaca mulatta *(1) and Viruses (3).

The current version of dbOGAP contains 1163 entries, including 798 experimentally determined O-GlcNAcylated proteins (Figure 2, *Left*, A), and 365 proteins with inferred O-GlcNAcylation sites (total 735 sites) based on O-GlcNAcylated orthologs (Figure 2, *Left*, D). About 22% of all known O-GlcNAcylated proteins (172/798) have O-GlcNAcylation sites identified (404 sites), among which 140 proteins also have known phosphorylation sites (1581 sites) as well as 357 O-GlcNAcylation sites (Figure 2, *Left*, B, C). Interestingly, 48 of those 140 proteins have 122 Ser/Thr sites that are potentially Yin-Yang sites to be subjected to possible reciprocal regulation by O-GlcNAcylation and phosohorylation, including 42 identical sites and 74 non-identical sites that are within 4 amino acids away from each other (Table 1). Overall, the number of currently identified O-GlcNAcylation sites is only ~11% (404/3687) of that of phosphorylation sites on all known O-GlcNAcylated proteins. Further, among all experimentally determined O-GlcNAcylated proteins, most (~61%) are of humans, and other organisms include rat (19.7%), mouse (8%), fruit fly (6.7%), and African frog (3.1%) (Figure 2, *Right*).

**Table 1:** O-GlcNAcylation and phosphorylation occurring at identical or adjacent (+/- 4 amino acids) serine/threonine (S/T) sites of O-GlcNAcylated proteins.

UniProt ID	Gene name	O-GlcNAcylation site	Identical phosphorylation S/T site	Adjacent phosphorylation S/T site
RRP1B_HUMAN	RRP1B	S731	S731	T728, S732, S735

CARF_HUMAN	CDKN2AIP	S348		T345

VIME_HUMAN	VIM	S7, T33, S34, S55	S7, T33, S34, S55	S5, S8, S9, S10, S29, T33, S34, S51, S56

SPTB2_HUMAN	SPTBN1	S2324		T2328

TPR_HUMAN	TPR	S1676		T1677

RBP2_HUMAN	RANBP2	T1399	T1399	T1396, S1400

H31_HUMAN	HIST1H3A	S11	S11	T12

K2C8_HUMAN	KRT8	S13, S15	S13	S9, S13, T14

MYC_HUMAN	MYC	T58	T58	S62

NUMA1_HUMAN	NUMA1	S1844		S1840, S1847

PHB_HUMAN	PHB	T258		S254

EMSY_HUMAN	EMSY	S236		S238

NU214_HUMAN	NUP214	T1201, S1354		T1203, S1356

CRTC2_HUMAN	CRTC2	S70, S171, S173	S70, S171	T169, S171, T177

KCC4_HUMAN	CAMK4	S356	S356	S360

FOXO1_HUMAN	FOXO1	T317		S319

BPTF_HUMAN	BPTF	T2094		S2098

HCFC1_HUMAN	HCFC1	T738	T738	T737

K1C18_HUMAN	KRT18	S30, S31, S49	S30, S31	S30, S31, S34, S47, S53

P121A_HUMAN	POM121	T693		S697

RBM14_HUMAN	RBM14	S244, S254, S256, S280	S256, S280	S256

AKT1_HUMAN	AKT1	T308, S473	S473, T308	

ATX2L_HUMAN	ATXN2L	S684	S684	

SYUA_HUMAN	SNCA	S87	S87	

IKKB_HUMAN	IKBKB	S733	S733	

ESR1_MOUSE	Esr1	S10	S10	T7

SPTB2_MOUSE	Sptbn1	S2323		T2327

BSN_MOUSE	Bsn	S1407, S2027, S2029, T2700, T2703	S2029, S2694, T2703	T1406, S2029, T2703

SYN1_MOUSE	Syn1	S518, T564		S520, S568

ABLM1_MOUSE	Ablim1	S496, S499	S496, S499	S494, T495, S496, S499, S502

SKT_MOUSE	Skt	S357		S359, S361

DEMA_MOUSE	Epb49	S285		S289

RBM14_MOUSE	Rbm14	S278		S280

CEBPB_MOUSE	Cebpb	S180, S181		S184

SRBS1_MOUSE	Sorbs1	S1199, S1200, S1201	S1201	S1201

ESR2_MOUSE	Esr2	S61	S61	

AKT1_MOUSE	Akt1	S473	S473	

NOS3_RAT	Nos3	S1178		T1174, S1176

SP1_RAT	Sp1	S613, T641, S642, S699, S703	S613, T641, S642, S703	T641, S642, S703

LBR_RAT	Lbr	S96		S99

TAU_RAT	Mapt	S711	S711	S707, T714, S715

KPCB_RAT	Prkcb	T635	T635	

KPCD_RAT	Prkcd	T295, T348	T295	S299

KPCE_RAT	Prkce	S368, T710	S368, T710	

KPCG_RAT	Prkcg	T689, S690		S687

SYN1_RAT	Syn1	S516, T562		S518, S566

G3P_RAT	Gapdh	T227	T227	

LT_SV40	SV40gp6	S111, S112	S112	S112

#### Functional profiles of O-GlcNAcylated proteins

We analyzed Gene Ontology (GO) profiles of currently known human O-GlcNAcylated proteins (~490) using the DAVID tool [29]. We first examined the major enriched GO categories of O-GlcNAcylated proteins annotated with GO terms at higher levels of GO hierarchy (covering ≥10% of the proteins) (Table 2). As shown by the GO *Cellular Components *profiling, O-GlcNAcylated proteins are mostly those of nucleoplasmic distribution, including membrane or non-membrane bounded organelles, cytosol, cytoskeleton, and nuclear compartments. The O-GlcNAcylated proteins mainly possess nucleotide and nucleic acid binding activities and transcription regulator activities (GO *Molecular Function*), and participate in transcriptional regulation, macromolecular complex assembly, intracellular transport, translation, regulation of cell cycle and apoptosis, and regulation of macromolecule metabolic process (GO *Biological Processes*).

**Table 2:** Major GO categories of human O-GlcNAcylated proteins.

Gene Ontology (GO) Terms	Count*	% Total	P-Value
**GO Biological Processes**			

GO:0045449~regulation of transcription	108	23.48	5.90E-04

GO:0006350~transcription	93	20.22	1.99E-04

GO:0051252~regulation of RNA metabolic process	75	16.30	6.09E-03

GO:0006355~regulation of transcription, DNA-dependent	69	15.00	3.08E-02

GO:0043933~macromolecular complex subunit organization	54	11.74	1.64E-09

GO:0065003~macromolecular complex assembly	53	11.52	4.68E-10

GO:0046907~intracellular transport	52	11.30	8.96E-10

GO:0007049~cell cycle	52	11.30	2.14E-07

GO:0006412~translation	51	11.09	3.30E-21

GO:0006396~RNA processing	51	11.09	4.00E-12

GO:0008104~protein localization	51	11.09	1.91E-05

GO:0010605~negative regulation of macromolecule metabolic process	48	10.43	1.39E-06

GO:0042981~regulation of apoptosis	48	10.43	1.60E-05

GO:0043067~regulation of programmed cell death	48	10.43	2.07E-05

GO:0010941~regulation of cell death	48	10.43	2.25E-05

GO:0045184~establishment of protein localization	47	10.22	1.11E-05

GO:0010604~positive regulation of macromolecule metabolic process	47	10.22	1.55E-04

**GO Molecular Function**			

GO:0000166~nucleotide binding	132	28.70	2.56E-13

GO:0003677~DNA binding	101	21.96	7.11E-04

GO:0032555~purine ribonucleotide binding	92	20.00	7.02E-06

GO:0032553~ribonucleotide binding	92	20.00	7.02E-06

GO:0017076~purine nucleotide binding	92	20.00	3.91E-05

GO:0030528~transcription regulator activity	83	18.04	6.87E-07

GO:0003723~RNA binding	82	17.83	2.05E-24

GO:0001882~nucleoside binding	82	17.83	1.56E-05

GO:0005524~ATP binding	81	17.61	1.03E-06

GO:0032559~adenyl ribonucleotide binding	81	17.61	1.73E-06

GO:0030554~adenyl nucleotide binding	81	17.61	1.26E-05

GO:0001883~purine nucleoside binding	81	17.61	2.16E-05

GO:0005198~structural molecule activity	56	12.17	8.96E-12

GO:0042802~identical protein binding	51	11.09	3.27E-09

**GO Cellular Component**			

GO:0043232~intracellular non-membrane-bounded organelle	170	36.96	6.37E-29

GO:0043228~non-membrane-bounded organelle	170	36.96	6.37E-29

GO:0005829~cytosol	141	30.65	7.16E-46

GO:0031974~membrane-enclosed lumen	131	28.48	6.60E-24

GO:0043233~organelle lumen	129	28.04	1.20E-23

GO:0070013~intracellular organelle lumen	128	27.83	5.00E-24

GO:0031981~nuclear lumen	116	25.22	2.44E-25

GO:0005856~cytoskeleton	77	16.74	2.21E-08

GO:0005654~nucleoplasm	77	16.74	2.07E-18

GO:0030529~ribonucleoprotein complex	72	15.65	3.55E-29

GO:0005730~nucleolus	55	11.96	3.09E-11

GO:0000267~cell fraction	49	10.65	2.01E-03

GO:0044430~cytoskeletal part	49	10.65	1.16E-04

* The table is sorted based on counts of proteins annotated with given GO terms in each of the major GO category. The percentage of proteins with given GO terms over total number of human O-GlcNAcylated proteins is shown (% Total). A more detailed GO profiles are shown in [Additional file 1, Supplementary Table S1].

We further examined the O-GlcNAcylated proteins for enrichment of GO terms at deeper levels of the GO hierarchy. As summarized in [Additional file 1, Supplementary Table S1], the top enriched GO *biological processes *relate to protein translation, carbohydrate (glucose) metabolism, RNA processing/splicing, and RNA/protein transport, followed by macromolecular complex and organelle organization, regulation of cell cycle and cell death, chromosome organization and transcription, regulation of protein and other small molecule metabolisms. The enriched GO *molecular functions *include nucleoside, nucleotide and nucleic acid binding, transcription factor activity, protein binding and other molecular activities. The enriched GO *cellular components *include cytosol, organelle lumen and non-membrane-bounded organelles, nuclear compartments such as nucleoplasm, nuclear pore and nucleolus, ribosome and cytoskeleton, nuclear protein complexes and chromatin, membrane and vesicle associated spaces, and contractile associated proteins. Notably, although significant proportions of known O-GlcNAcylated proteins are associated with intracellular membranes or inner side of plasma membrane, only a few plasma transmembrane proteins, such as glucose transporters and notch receptor were reported to be O-GlcNAcylated [30-32]. Therefore O-GlcNAcylated proteins are primarily nucleocytoplasmic and are engaged in broad biological functions.

#### Pathways and disease processes related to O-GlcNAcylated proteins

We examined pathway profiles of O-GlcNAcylated human proteins using GeneGO Pathway Maps [33]. A wide range of cellular pathways contain significant numbers of proteins that are known to be subjected to O-GlcNAcylation, including pathways involved in growth, development and differentiation, immune and inflammatory responses, cytoskeleton remodeling, and metabolic pathways such as gluconeogenesis. A total of 141 annotated GeneGO pathways are significantly enriched for the O-GlcNAcylated human proteins (p-value < 1.0E-03). Table 3 lists 42 pathways that are enriched at a p-value of <1.0E-05, many of which are growth factor signaling (e.g., EGFR, VEGF, TGFβ, and AKT) and cytokine signaling (e.g., GM-CSF, IL-2, IL6/7) pathways.

**Table 3:** Pathway profiles using GeneGo Pathway Maps analysis.

Pathways	P-value	Count*
Development_Role of CDK5 in neuronal development	2.68E-11	12/34

Development_Gastrin in cell growth and proliferation	3.86E-11	15/62

Immune response_Gastrin in inflammatory response	2.00E-10	15/69

Signal transduction_Activation of PKC via G-Protein coupled receptor	5.21E-10	13/52

Cytoskeleton remodeling_Cytoskeleton remodeling	9.88E-10	17/102

Glycolysis and gluconeogenesis (short map)	1.43E-09	14/67

Cytoskeleton remodeling_Neurofilaments	7.28E-09	9/25

Transcription_Role of Akt in hypoxia induced HIF1 activation	1.59E-08	9/27

Immune response_MIF - the neuroendocrine-macrophage connector	1.93E-08	11/46

Development_Prolactin receptor signaling	2.50E-08	12/58

Cytoskeleton remodeling_TGF, WNT and cytoskeletal remodeling	2.72E-08	16/111

Development_Gastrin in differentiation of the gastric mucosa	3.22E-08	10/38

Development_GM-CSF signaling	4.94E-08	11/50

Development_EGFR signaling pathway	6.66E-08	12/63

Cytoskeleton remodeling_Regulation of actin cytoskeleton by Rho GTPases	7.06E-08	8/23

G-protein signaling_Proinsulin C-peptide signaling	7.62E-08	11/52

Development_Glucocorticoid receptor signaling	1.04E-07	8/24

Development_VEGF signaling and activation	1.16E-07	10/43

Cell adhesion_Histamine H1 receptor signaling in the interruption of cell barrier integrity	1.85E-07	10/45

Immune response_Inhibitory action of Lipoxins on pro-inflammatory TNF-alpha signaling	1.85E-07	10/45

Signal transduction_Calcium signaling	1.85E-07	10/45

Regulation of CFTR activity (norm and CF)	2.50E-07	11/58

Translation _Regulation of translation initiation	2.92E-07	8/27

Immune response_Histamine H1 receptor signaling in immune response	3.53E-07	10/48

Immune response_IL-2 activation and signaling pathway	4.34E-07	10/49

Transcription_P53 signaling pathway	5.48E-07	9/39

Development_Slit-Robo signaling	7.19E-07	8/30

Development_Ligand-dependent activation of the ESR1/AP-1 pathway	7.81E-07	6/14

Development_PDGF signaling via STATs and NF-kB	1.24E-06	8/32

Signal transduction_AKT signaling	1.33E-06	9/43

Development_VEGF signaling via VEGFR2 - generic cascades	2.00E-06	9/45

Development_Thrombopoietin-regulated cell processes	2.00E-06	9/45

Mucin expression in CF via IL-6, IL-17 signaling pathways	2.04E-06	8/34

Development_TGF-beta-dependent induction of EMT via RhoA, PI3K and ILK.	2.43E-06	9/46

Development_PIP3 signaling in cardiac myocytes	2.93E-06	9/47

Cytoskeleton remodeling_Keratin filaments	3.24E-06	8/36

Development_Thyroliberin signaling	3.61E-06	10/61

Development_A3 receptor signaling	4.22E-06	9/49

Transport_RAN regulation pathway	4.42E-06	6/18

Immune response_IL-7 signaling in T lymphocytes	5.01E-06	8/38

Muscle contraction_Regulation of eNOS activity in endothelial cells	5.66E-06	10/64

Immune response_IL-6 signaling pathway	7.57E-06	7/29

*Number of known O-GlcNAcylated proteins over the total number of proteins annotated in the corresponding GeneGO pathways. The table is ranked based on the pathway enrichment P-values.

Because of the broad cellular processes and pathways that the O-GlcNAcylated proteins are known to participate in, O-GlcNAcylation may potentially play significant roles in many pathological conditions. Indeed, four categories of disease conditions have been implicated to involve O-GlcNAcylation, i.e., type II diabetes, neurodegenerative diseases, cardiovascular diseases, and cancers [34]. For example, OGT regulates insulin signaling through O-GlcNAcylation of several important insulin signaling molecules, e.g., IRS-1, PI3K, PDK1, and AKT1, leading to attenuation of insulin signaling responses in glycogen synthesis, activation of gluconeogenic genes and glucose transporter GLUT4 translocation, thus contributing to insulin resistance in type II diabetes [1,35]. Tau protein is subject to both OGlcNAcylation and phosphorylation, and hyperphosphorylation apparently contributes to neurofibrillary tangle formation by tau in Alzheimer's disease [36]. O-GlcNAcylation represents a key regulatory mechanism in modulating vascular reactivity, such as contractile and relaxant response through modification of proteins, e.g., NOS, sarcoplasmic reticulumn Ca(2+)-ATPase, PKC, MAPK and cytoskeleton and microtubule proteins [37]. O-GlcNAcylation can mediate cardiac stress responses and has cardioprotective effects through transcription-mediated regulation as well as cardiomyocyte calcium homeostasis [38]. O-GlcNAcylation may have general roles in cancer through its involvement in oncogenesis or tumor suppression by coupling cellular metabolic status to regulation of signal transduction, transcription, and protein degradation. For example, reducing cellular UDP-GlcNAc level in MCF-7 cells changed the O-GlcNAcylation patterns of key proteins that control cell proliferation and differentiation, including Sp1, chaperonin TCP-1 theta, and oncogene Ets-related protein isoform 7 [39]. Many cancer genes or tumor suppressors are known to be O-GlcNAcylated or to interact with OGT, such as c-Myc, AKT1, AKT2, ATF1, CBP, FOXO1, TOP1, p53 and HIC1 [40]. In breast cancer cells, knockdown of OGT and the resulting global reduction of O-GlcNAcylation inhibited cell proliferation and metastasis ability [41].

### 2. The O-GlcNAcylation Site Prediction

**Figure 3:**
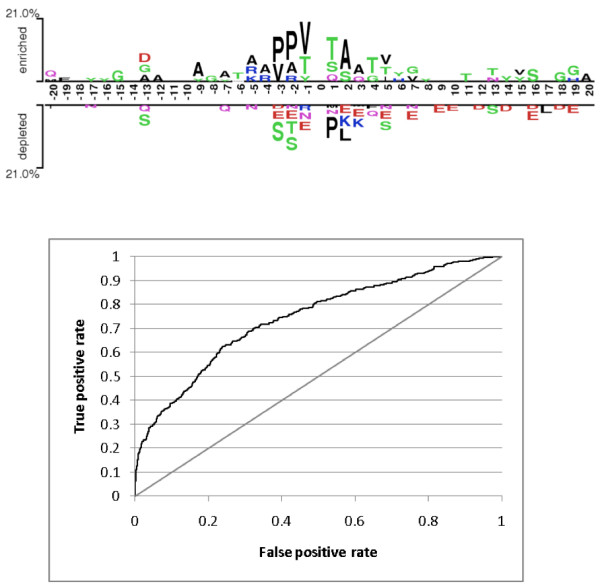
**Sequence patterns and prediction performance of O-GlcNAcylation sites.*** Above*, Graphical representation of sequence patterns surrounding the O-GlcNAcylation sites as determined by *Two Sample Logo*. The height of the amino acid character represents the relative frequency (enrichment or depletion) of the amino acid at any given positions relative to the O-GlcNAcylated residue (S/T at position "0"). *Below*, An ROC curve of OGlcNAcScan obtained in a five-fold cross-validation test. The area under this curve (i.e., AUC) is 74.3% of the plot area. The diagonal line indicates the ROC curve of random guessing, where the corresponding AUC value is 50%.

Figure 3 (*Above*) shows the graphical representation of sequence patterns surrounding the O-GlcNAcylation sites annotated in dbOGAP using the "Two Sample Logo" tool [42]. Enrichment of amino acids at -3/+2 position of the modified Ser/Thr, PPV(S/T)TA, can be observed. However, the amino acid enrichment at each position independently is not sufficient for defining a sequence motif for O-GlcNAcylation sites. OGlcNAcScan was designed to exploit sequence properties through SVM for the site prediction. The system achieved an *area under ROC (the receiver operating characteristic) curve *(AUC) of 74.3% (Figure 3, *Below*) in a five-fold cross-validation test. AUC is a widely used performance measure of binary classifiers. A perfect classifier yields an AUC of 100% while random guessing yields that of 50%. Although the AUC value of OGlcNAcScan is relatively low, we need to consider at least the following two factors for its interpretation. First, the fraction of positive instances is extremely low in this task, i.e., 373 (1.23%) of 30270 Ser/Thr sites are annotated O-GlcNAcylation sites in dbOGAP. Some of the past studies on PTM site prediction reported the performance of prediction systems on a balanced data set, where sampled negative sites were used in the evaluation data set (e.g., the ratio of positive and negative sites were made to be 1:1 (50% positive) or 1:5 (16.7% positive)). In fact, the relative improvement of our trained SVM classifier, when compared to random guessing [43], can be as high as 14-fold (i.e., the *precision *of the classifier can be 14 times higher than the original rate of positives sites of 1.23%). The second factor to be considered is that negative instances in the evaluation data set may include not-yet-annotated true O-GlcNAcylation sites, which could have lowered the performance measures. We believe, however, sequence-based prediction of O-GlcNAcylation sites is inherently challenging. Additional training data through further annotation of proteins and sites as well as incorporation of other feature types, such as physiochemical properties of amino acids and protein structure information, may help improve the performance.

### 3. The dbOGAP Web Site

**Figure 4:**
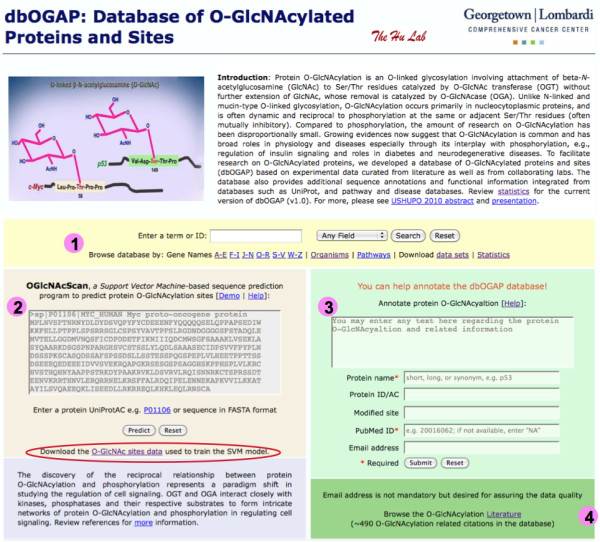
**The dbOGAP website home page**. The website provides functionalities depicted by #1-#4: 1) search and browse the O-GlcNAcylated proteins in the database; 2) de novo prediction of O-GlcNAcylation sites for any protein sequences; 3) user annotation of O-GlcNAcylation information; 4) search and browse the total O-GlcNAcylation bibliography. The dbOGAP web site can be accessed at http://cbsb.lombardi.georgetown.edu/OGAP.html.

The dbOGAP web site provides two primary functionalities, search, query and browse of O-GlcNAcylated proteins and their related annotations, and *de novo *prediction of O-GlcNAcylation sites (Figure 4, #1 and #2). The dbOGAP database can be searched based on gene/protein names or identifiers, pathway names, or PubMed IDs. The protein entries can also be browsed based on gene names, organisms or pathways. The OGlcNAcScan site prediction system allows input of a protein sequence in FASTA format or a UniProtKB identifier (AC or ID) for site prediction. In addition, users can contribute their annotations to the database based on literature or from unpublished proteomic data on newly identified O-GlcNAcylation sites (Figure 4, #3). All O-GlcNAcylation related literature citations are also available for browsing (Figure 4, #4).

#### The O-GlcNAcylated protein entry

**Figure 5:**
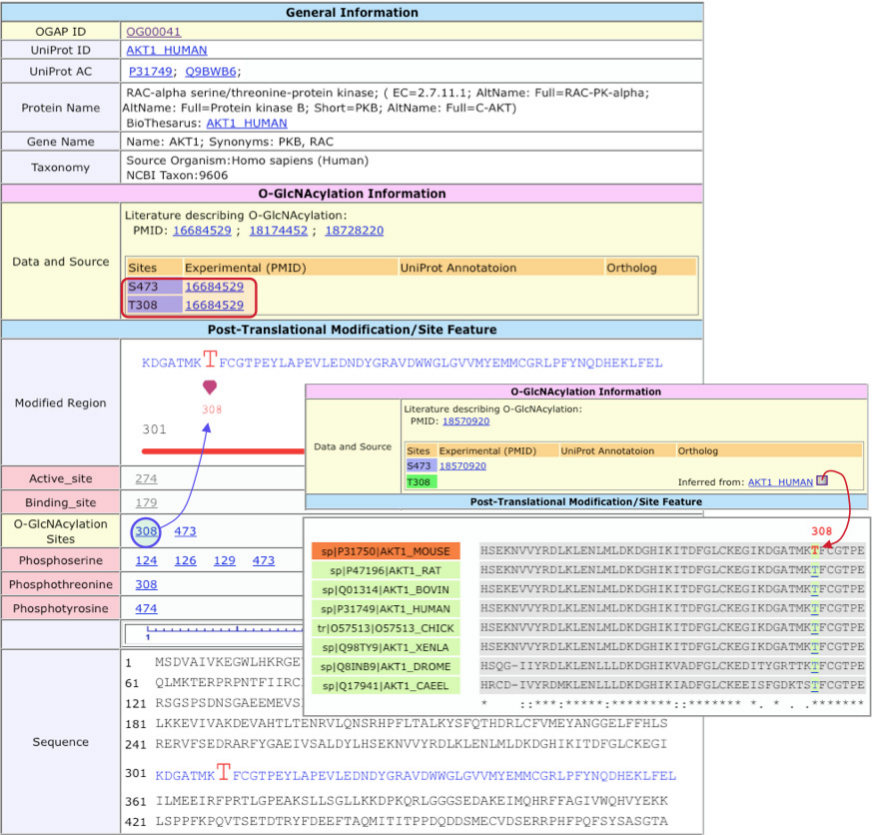
**The dbOGAP protein entry view (shown is human AKT1)**. The entry report provides general protein information as well as specific O-GlcNAcylation information in the context of other posttranslational modifications and site features. The literature evidence (PMID) for the O-GlcNAc sites (e.g. S473 and T308) is given. Clicking on any site will display the residue in the neighboring sequence context (pointed by blue arrow). If the O-GlcNAcylation sites are inferred from orthologs with known sites (e.g. T308 of mouse AKT1, pointed by red arrow, inferred from human AKT1 shown in the inset), sequence alignment for the inferred sites can be displayed (lower portion of the inset). Other annotations are also included in the entry record (below the sequence section, not shown), including gene ontology, pathway, derived from UniProtKB and iProClass.

The dbOGAP protein entries are assigned unique IDs (e.g., OG00001) and are mapped to the corresponding UniProtKB IDs (1433B_HUMAN) and Accessions (P31946). The entry report provides detailed O-GlcNAcylation information and evidence attributions, including experimental and inferred O-GlcNAcylaytion data (Figure 5). O-GlcNAcylated residues and positions, as well as other modification sites (e.g., phosphorylation) and site features (e.g., binding sites), can be visualized in the context of protein sequences. The entry record also provides additional annotations such as GO, pathways (e.g., KEGG, PID and Reactome), protein-protein interactions (e.g., IntAct), protein families (e.g., Pfam) and diseases (OMIM), as well as additional protein bibliography integrated from UniProt and iProClass. Hyperlinks to source databases are provided for integrated annotations in dbOGAP entry records.

#### The O-GlcNAcScan report

**Figure 6:**
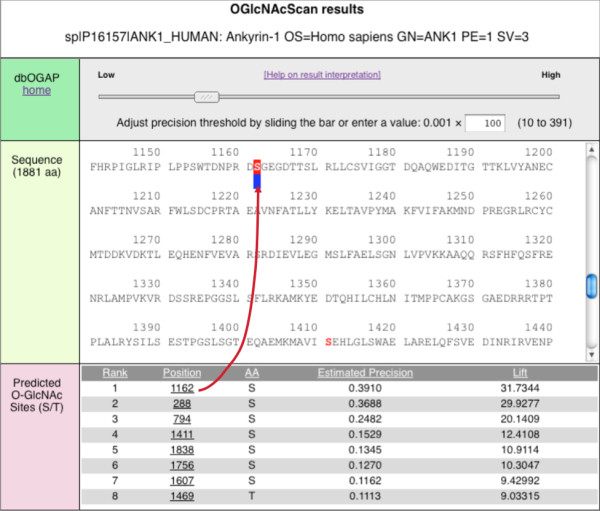
**The O-GlcNAcylation site prediction result from OGlcNAcScan (shown is human ankyrin-1)**. The section at the bottom displays a ranked list of predicted O-GlcNAcylation sites (e.g., S1162 as the top one). The rank is based on the output value of the SVM classifier, which is converted into "Estimated Precision" and "Lift" scores (see help page linked from the top of the page for explanation). The estimated precision score is an estimated lower-bound of the precision (e.g., the score of 0.3910 indicates that at least 39.1% of sites assigned with the similar SVM output scores are O-GlcNAcylation sites), and the Lift score is an index of relative improvement through the classifier, which is calculated as the estimated precision divided by a constant value corresponding to the initial rate of positive sites (i.e., ~0.0123). All displayed potential sites are shown as red "S/T" in the sequence section (middle). Clicking on any predicted site, the residue will be highlighted in the sequence (arrow).

The OGlcNAcScan report page provides a list of predicted O-GlcNAcylation sites for a given query sequence (Figure 6). The list can be sorted based on the prediction scores, positions of predicted sites, and the amino acids. The predicted site of interest can be highlighted in the protein sequence. The threshold for display of O-GlcNAcylation sites can be adjusted to increase or reduce the number of predictions. More detailed explanation and interpretation of the prediction results are provided in the online help document.

#### The dbOGAP database download

The dbOGAP web site provides a download page (Figure 4, linked in #1) for downloading the database in several data files, including all O-GlcNAcylated proteins, sites and orthologs. A full bibliography of O-GlcNAcylated proteins can also be downloaded. The data sets for developing the OGlcNAcScan system are available to the scientific community for further development of the site prediction (Figure 4, #2).

## Discussion

Up to now, the amount of data published on protein O-GlcNAcylation is only a fraction of that of phosphorylation, and its biological role is much less understood. Since 2006, the identification of O-GlcNAcylated proteins and sites has been rapidly growing due to the improved mass spectrometry technologies and O-GlcNAc enrichment techniques [7-9]. The dbOGAP database provides a timely bioinformatics resource to allow readily access by the community to the known and potential O-GlcNAcylated proteins and sites.

While a large number of O-GlcNAcylated proteins and sites were identified in recent years, many were determined based on large-scale mass spectrometry and would need to be further validated. Although O-GlcNAcylation has been known to occur primarily in nucleocytoplamic proteins, the GO profiles show that O-GlcNAcylated proteins are localized in a broad range of intracellular compartments. Interestingly, some O-GlcNAcylated proteins are of unusual classes, e.g., adenylate kinase 2 (AK2, UniProtKB: KAD2_HUMAN) [44] localized in the mitochondria inter-membrane space, and alpha-1-inhibitor 3 (A1i3, UniProtKB: A1I3_RAT) [45], a secreted protein. Although false positive identification of O-GlcNAcylation is not uncommon from mass spectrometry, it is possible that such proteins may be indeed O-GlcNAcylated. It is known that OGT has at least three isoforms differing in N-terminal sequences with identical catalytic domain, the mitochondrial (mOGT) and two nucleocytoplasmic forms (ncOGT and sOGT) [46,47]. The mOGT form was shown associated with the mitochondrial inner membrane [46], thus consistent with the observation of O-GlcNAcylation of the mitochondrial protein AK2. There are a total of ~11 O-GlcNAcylated proteins in dbOGAP that are known to be secreted or have secreted forms besides cytoplasmic ones. It is possible that only the cytoplasmic forms of some of these proteins are O-GlcNAcylated while the secreted ones may not, albeit experimental validation is needed. Thus, the types and/or sources of O-GlcNAcylation identification have been assigned to protein entries as evidence attribution to annotations in the dbOGAP database.

The OGlcNAcScan site prediction system provides a much needed tool for studying protein glycosylation as well as phosphorylation. Since the site prediction is primarily based on the protein sequence context, some secreted proteins may be erroneously predicted even with a relatively high score, e.g., T298 in mucin 4 (UniProtKB: MUC4_HUMAN) predicted with a score of 0.287, though it is unlikely to be O-GlcNAcylated. In such cases, a cautionary note is given to indicate that a protein sequence being predicted is known to have "secreted" form(s). With the continuing growth of O-GlcNAcylation sites data, the OGlcNAcScan tool will be further enhanced through retraining the SVM model, as well as by integrating physiochemical properties and structural information into the SVM prediction model.

## Conclusion

In conclusion, the dbOGAP database and the web site become the first of its kind in the public domain to provide readily access to a curated and systematic collection of protein O-GlcNAcylation information, and to a state-of-the-art O-GlcNAcylation site prediction system, OGlcNAcScan, to assist proteomic identification of O-GlcNAc modification sites. Thus, the dbOGAP resource should benefit the biological community to study the broad roles of O-GlcNAcylation in physiology and diseases.

## Availability and Requirements

The dbOGAP database and the OGlcNAcScan system can be publicly accessed at: http://cbsb.lombardi.georgetown.edu/OGAP.html. The database and related data sets can be downloaded at: http://cbsb.lombardi.georgetown.edu/filedown.php.

## Authors' contributions

JW is responsible for the design and implementation of the database and the web site development for most of the web pages. MT is responsible for developing the OGlcNAcScan system and the web display of the prediction results. HL contributed to the machine learning methods for OGlcNAcScan and to the design and testing of the dbOGAP web interface. GWH contributed experimental O-GlcNAcylation data for populating the database. ZZH conceived the overall design of the database and site prediction, and is responsible for the O-GlcNAcylation data curation from literature and the web site testing. All authors read and approved the manuscript.

## Supplementary Material

Additional file 1 **Supplementary Table S1. Major categories of O-GlcNAcylated proteins based on GO terms at deeper level of GO hierarchy**. This table provides GO profiles at deeper level of GO terms to complement the major GO profiles of O-GlcNAcylated proteins in Table 2. Click here for file
