# Supplementary material for: dbOGAP - An Integrated Bioinformatics Resource for Protein O-GlcNAcylation
Source: BMC Bioinformatics. 2011 Apr 6;12:91. doi: 10.1186/1471-2105-12-91 (PMC3083348; doi:10.1186/1471-2105-12-91)
Supplement: Additional file 1 — Supplementary Table S1. Major categories of O-GlcNAcylated proteins based on GO terms at deeper level of GO hierarchy. This table provides GO profiles at deeper level of GO terms to complement the major GO profiles of O-GlcNAcylated proteins in Table 2. [file 1471-2105-12-91-S1.DOC]

**ADDITIONAL MATERIAL**

**Supplementary Table S1**. Major categories of O-GlcNAcylated proteins based on GO terms at deeper level of GO hierarchy.

| **Gene Ontology (GO) Terms** | **Count** | **P-Value*** |
| --- | --- | --- |
| **GO Biological Process** | | |
| **Protein translation and amino acid metabolism** |  |  |
| GO:0006412~translation | 51 | 3.30E-21 |
| GO:0006414~translational elongation | 28 | 1.93E-18 |
| GO:0043039~tRNA aminoacylation | 10 | 9.05E-06 |
| GO:0043038~amino acid activation | 10 | 9.05E-06 |
| **Carbohydrate metabolism** |  |  |
| GO:0006007~glucose catabolic process | 18 | 8.84E-13 |
| GO:0006096~glycolysis | 16 | 4.95E-12 |
| GO:0019320~hexose catabolic process | 18 | 1.96E-11 |
| GO:0016052~carbohydrate catabolic process | 20 | 7.76E-10 |
| **RNA processing, splicing, metabolism and stability** |  |  |
| GO:0006396~RNA processing | 51 | 4.00E-12 |
| GO:0008380~RNA splicing | 35 | 1.02E-11 |
| GO:0016071~mRNA metabolic process | 39 | 6.24E-11 |
| GO:0043488~regulation of mRNA stability | 7 | 4.00E-05 |
| **Nucleic acid and protein transport and localization** |  |  |
| GO:0051236~establishment of RNA localization | 21 | 1.12E-11 |
| GO:0050658~RNA transport | 21 | 1.12E-11 |
| GO:0034613~cellular protein localization | 37 | 1.52E-08 |
| GO:0006886~intracellular protein transport | 33 | 1.70E-07 |
| **Macromolecular complex and organelle organization** |  |  |
| GO:0065003~macromolecular complex assembly | 53 | 4.68E-10 |
| GO:0006461~protein complex assembly | 41 | 3.92E-08 |
| GO:0007010~cytoskeleton organization | 33 | 4.86E-06 |
| GO:0033043~regulation of organelle organization | 20 | 4.15E-05 |
| **Cell cycle and cell death regulation** |  |  |
| GO:0007049~cell cycle | 52 | 2.14E-07 |
| GO:0022402~cell cycle process | 40 | 1.99E-06 |
| GO:0043066~negative regulation of apoptosis | 28 | 1.37E-05 |
| GO:0006916~anti-apoptosis | 20 | 2.01E-05 |
| **Chromosome organization and transcription regulation** |  |  |
| GO:0006325~chromatin organization | 29 | 1.62E-05 |
| GO:0051276~chromosome organization | 34 | 1.69E-05 |
| GO:0006357~regulation of transcription from RNA polymerase II promoter | 43 | 6.06E-05 |
| GO:0045893~positive regulation of transcription, DNA-dependent | 32 | 7.20E-05 |
| **Protein metabolic process** |  |  |
| GO:0051248~negative regulation of protein metabolic process | 22 | 3.03E-07 |
| GO:0010605~negative regulation of macromolecule metabolic process | 48 | 1.39E-06 |
| GO:0032269~negative regulation of cellular protein metabolic process | 20 | 2.84E-06 |
| GO:0031400~negative regulation of protein modification process | 15 | 1.70E-05 |
| **Other metabolism** |  |  |
| GO:0046164~alcohol catabolic process | 18 | 3.00E-10 |
| GO:0051173~positive regulation of nitrogen compound metabolic process | 43 | 3.36E-06 |
| GO:0006091~generation of precursor metabolites and energy | 25 | 3.78E-05 |
| GO:0043603~cellular amide metabolic process | 10 | 4.74E-05 |
| **GO Molecular Function** | | |
| **Nucleoside, nucleotide binding** |  |  |
| GO:0000166~nucleotide binding | 132 | 2.56E-13 |
| GO:0005524~ATP binding | 81 | 1.03E-06 |
| GO:0032559~adenyl ribonucleotide binding | 81 | 1.73E-06 |
| GO:0001882~nucleoside binding | 82 | 1.56E-05 |
| **RNA binding** |  |  |
| GO:0003723~RNA binding | 82 | 2.05E-24 |
| GO:0008135~translation factor activity, nucleic acid binding | 15 | 2.49E-06 |
| GO:0003727~single-stranded RNA binding | 7 | 1.68E-04 |
| GO:0003729~mRNA binding | 10 | 2.27E-04 |
| **DNA binding** |  |  |
| GO:0003690~double-stranded DNA binding | 16 | 3.83E-07 |
| GO:0003697~single-stranded DNA binding | 11 | 7.73E-06 |
| GO:0042162~telomeric DNA binding | 6 | 7.33E-05 |
| GO:0010843~promoter binding | 9 | 4.13E-04 |
| **Transcription factor activity** |  |  |
| GO:0016563~transcription activator activity | 37 | 3.33E-08 |
| GO:0030528~transcription regulator activity | 83 | 6.87E-07 |
| GO:0003713~transcription coactivator activity | 18 | 4.93E-04 |
| GO:0016564~transcription repressor activity | 23 | 4.97E-04 |
| **Protein binding** |  |  |
| GO:0051082~unfolded protein binding | 23 | 9.68E-12 |
| GO:0019899~enzyme binding | 44 | 1.02E-08 |
| GO:0008022~protein C-terminus binding | 18 | 2.40E-06 |
| GO:0008092~cytoskeletal protein binding | 35 | 2.95E-05 |
| **Other** |  |  |
| GO:0005198~structural molecule activity | 56 | 8.96E-12 |
| GO:0004812~aminoacyl-tRNA ligase activity | 10 | 1.42E-05 |
| GO:0003746~translation elongation factor activity | 6 | 8.19E-04 |
| GO:0030235~nitric-oxide synthase regulator activity | 4 | 3.05E-04 |
| **GO Cellular Component** | | |
| **Cytosol** |  |  |
| GO:0005829~cytosol | 141 | 7.16E-46 |
| GO:0044445~cytosolic part | 31 | 4.28E-17 |
| GO:0005625~soluble fraction | 30 | 3.39E-08 |
| GO:0048471~perinuclear region of cytoplasm | 20 | 7.56E-04 |
| **Organelle lumen and non-membrane-bounded organelle** |  |  |
| GO:0043228~non-membrane-bounded organelle | 170 | 6.37E-29 |
| GO:0031974~membrane-enclosed lumen | 131 | 6.60E-24 |
| GO:0070013~intracellular organelle lumen | 128 | 5.00E-24 |
| GO:0043233~organelle lumen | 129 | 1.20E-23 |
| **Nuclear compartments** |  |  |
| GO:0031981~nuclear lumen | 116 | 2.44E-25 |
| GO:0005654~nucleoplasm | 77 | 2.07E-18 |
| GO:0005643~nuclear pore | 20 | 7.07E-13 |
| GO:0005730~nucleolus | 55 | 3.09E-11 |
| GO:0005635~nuclear envelope | 24 | 2.84E-08 |
| **Ribosome** |  |  |
| GO:0022626~cytosolic ribosome | 21 | 1.01E-13 |
| GO:0005840~ribosome | 30 | 4.48E-12 |
| GO:0022625~cytosolic large ribosomal subunit | 11 | 8.61E-08 |
| GO:0022627~cytosolic small ribosomal subunit | 9 | 1.59E-05 |
| **Cytoskeleton** |  |  |
| GO:0030863~cortical cytoskeleton | 15 | 6.34E-11 |
| GO:0005938~cell cortex | 22 | 1.32E-09 |
| GO:0005856~cytoskeleton | 77 | 2.21E-08 |
| GO:0015630~microtubule cytoskeleton | 36 | 1.05E-05 |
| **Ribonucleoprotein, nuclear protein complexes and chromatin** |  |  |
| GO:0030529~ribonucleoprotein complex | 72 | 3.55E-29 |
| GO:0030530~heterogeneous nuclear ribonucleoprotein complex | 10 | 2.63E-10 |
| GO:0005681~spliceosome | 18 | 2.61E-07 |
| GO:0044427~chromosomal part | 30 | 2.76E-06 |
| **Membrane and vesicle associated spaces** |  |  |
| GO:0046930~pore complex | 20 | 2.36E-11 |
| GO:0030120~vesicle coat | 10 | 1.03E-06 |
| GO:0012505~endomembrane system | 42 | 1.67E-04 |
| GO:0009898~internal side of plasma membrane | 21 | 9.27E-04 |
| **Contractile associated proteins** |  |  |
| GO:0005832~chaperonin-containing T-complex | 5 | 2.28E-05 |
| GO:0030017~sarcomere | 13 | 2.57E-05 |
| GO:0030016~myofibril | 13 | 8.89E-05 |
| GO:0043292~contractile fiber | 13 | 2.03E-04 |

* The GO terms are sorted in each category based on the enrichment p-value.
